# Supplementary material for: Trisomy 21 activates the kynurenine pathway via increased dosage of interferon receptors
Source: Nat Commun. 2019 Oct 18;10:4766. doi: 10.1038/s41467-019-12739-9 (PMC6800452; doi:10.1038/s41467-019-12739-9)
Supplement: Supplementary file 1 — Supplementary Information [file 41467_2019_12739_MOESM1_ESM.pdf]

Supplementary Information for:

Trisomy 21 activates the kynurenine pathway via increased dosage of interferon receptors

Rani K. Powers, Rachel Culp-Hill, Michael P. Ludwig, Keith P. Smith, Katherine A. Waugh, Ross Minter, Kathryn D. Tuttle, Hannah C. Lewis, Angela L. Rachubinski, Ross E. Granrath, Carmona-Iragui, Rebecca B. Wilkerson, Darcy E. Kahn, Molishree Joshi, Alberto Lleó, Rafael Blesa, Juan Fortea, Angelo D'Alessandro, James C. Costello, Kelly D. Sullivan, & Joaquin M. Espinosa

Supplementary Figures.

Powers et al., Supplementary Fig. 1

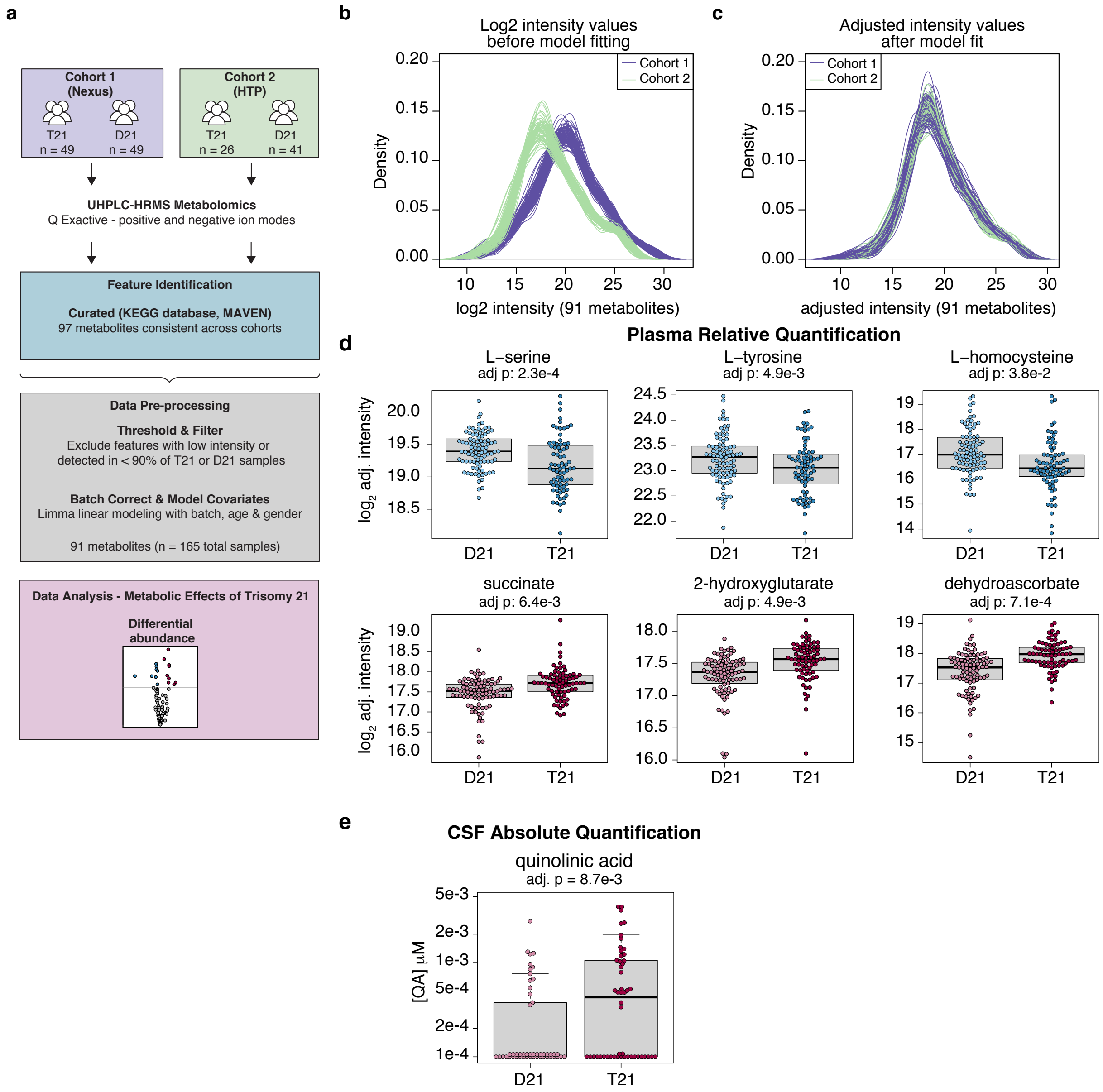

Supplementary Figure 1 - Statistical workflow adjusts for the effects of age and sex in circulating plasma metabolites.

a) Overview of the analysis pipeline used to identify, annotate, and analyze plasma metabolites measured in individuals with Down syndrome (T21 group) and controls without Down syndrome (D21) from the Translational Nexus Biobank (Nexus, Cohort 1, n=98, 49 with T21) and the Human Trisome Project (HTP, Cohort 2, n=67, 26 with T21). Putative metabolites were

annotated using MAVEN. Across the two cohorts, 97 metabolites were detected consistently. Subsequent data preprocessing steps included filtering out metabolites with low intensity values (per-metabolite threshold informed by blanks) or that were detected in less than 90% of the T21 samples or less than 90% of the D21 samples, leaving 91 metabolites. Batch correction and linear model fitting was performed before the combined data set was used for analysis. **b)** Density plots showing the distribution of $\log_2$ intensity values for all 91 metabolites in Cohort 1 (purple) and Cohort 2 (green) prior to any adjustment. **c)** Density plots showing the distribution of adjusted $\log_2$ intensity values for all 91 metabolites, colored as in **b**, after a linear model was used to adjust for age, sex, and cohort covariates. **d)** Boxplots showing $\log_2$ adjusted intensities for significantly differentially abundant metabolites. p-values were calculated using the linear model from **a** and the FDR method for multiple testing correction, n=165 independent samples, 75 with T21. **e)** Boxplot of quinolinic acid levels from CSF using the absolute quantification method, n=100 independent samples, 50 with T21. All boxplots show median, 25th, and 75th percentile values. Error bars are 1.5 times the interquartile range (IQR) or the maximum data point if less than 1.5 IQR.

a

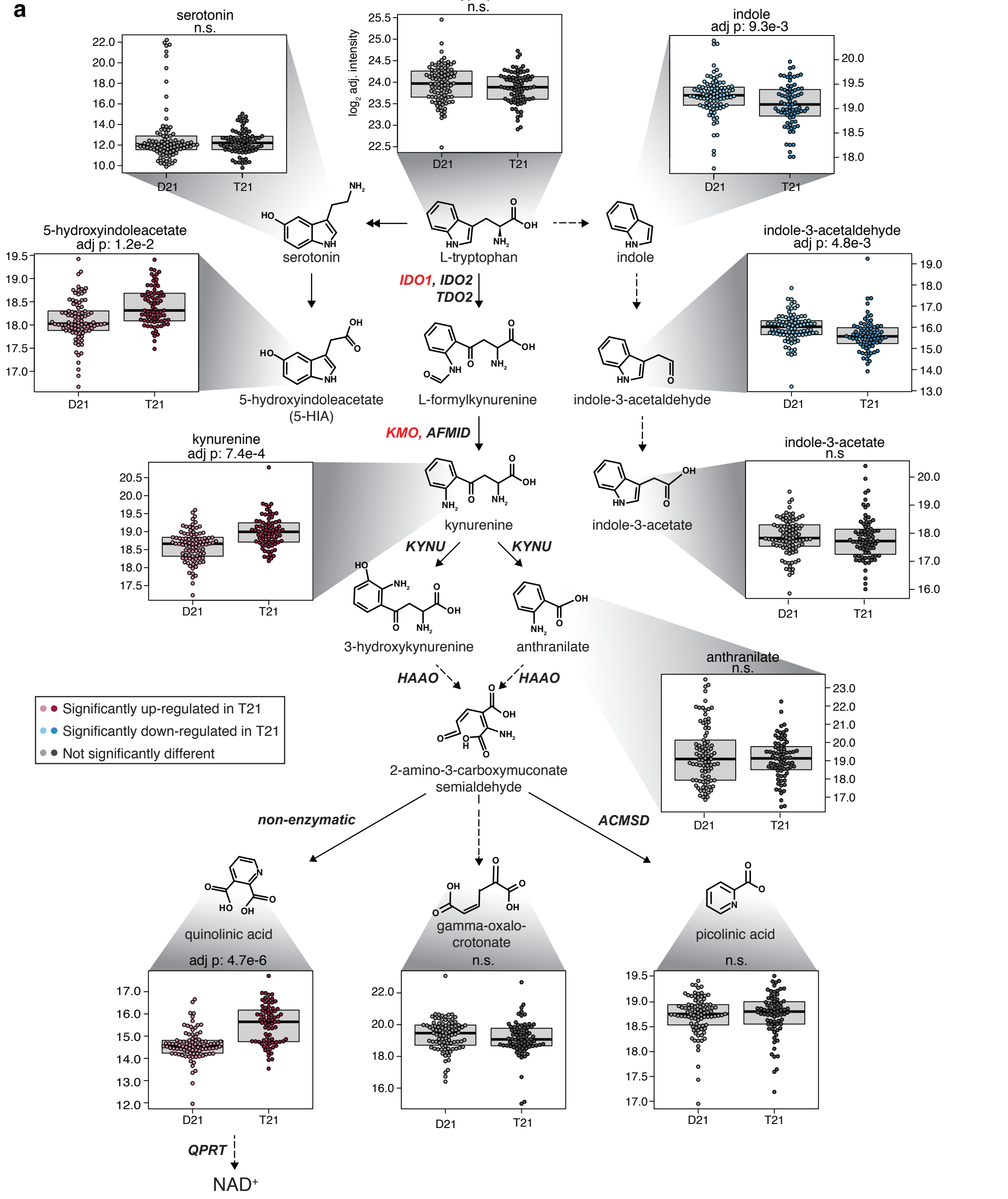

b

RNAseq - white blood cells

Gene	log_2foldchange	adj p
IDO1	1.24	8.04e-5
IDO2	1.27	3.85e-1
TDO2	-	-
KMO	-0.58	7.71e-4
AFMID	0.47	1.11e-1
KYNU	0.00	9.97e-1
HAAO	-0.41	3.32e-1
QPRT	0.24	5.30e-1
ACMSD	-	-
KYAT1	-	-
AADAT	-	-
KYAT3	-	-

c

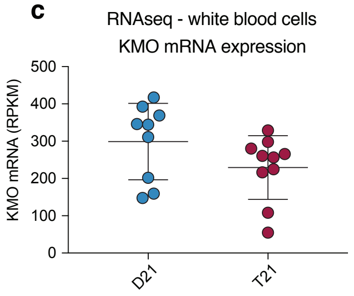

Supplementary Figure 2 – Trisomy 21 induces the kynurenine pathway. a) Schematic of tryptophan metabolism and the kynurenine pathway. Metabolite levels are plotted as adjusted intensity after adjusting for age, sex, and cohort (see Methods). p-values were calculated using the linear model from **Figure 1a** with FDR for multiple testing correction from Cohorts 1 and 2, n=165 independent samples, 75 with T21. Multi-headed arrows are used to represent multiple steps in the pathway that are not shown. Arrows with dashed lines represent reactions occurring in tryptophanase-expressing gastrointestinal microbiota. **b)** Table displaying fold change and significance level for various enzymes in the kynurenine pathway. Data are derived from RNA-seq of white blood cells from individuals with trisomy 21 and controls. Absent values indicate no detectable mRNA expression. **c)** Scatter plot showing RNA expression of *KMO* in white blood cells from Cohort 5, n=19 independent samples, 10 with T21. Statistical significance was calculated using DESeq2. mRNA expression values are displayed in reads per kilobase per million (RPKM)**.** All boxplots show median, 25th, and 75th percentile values. Error bars are standard deviation.

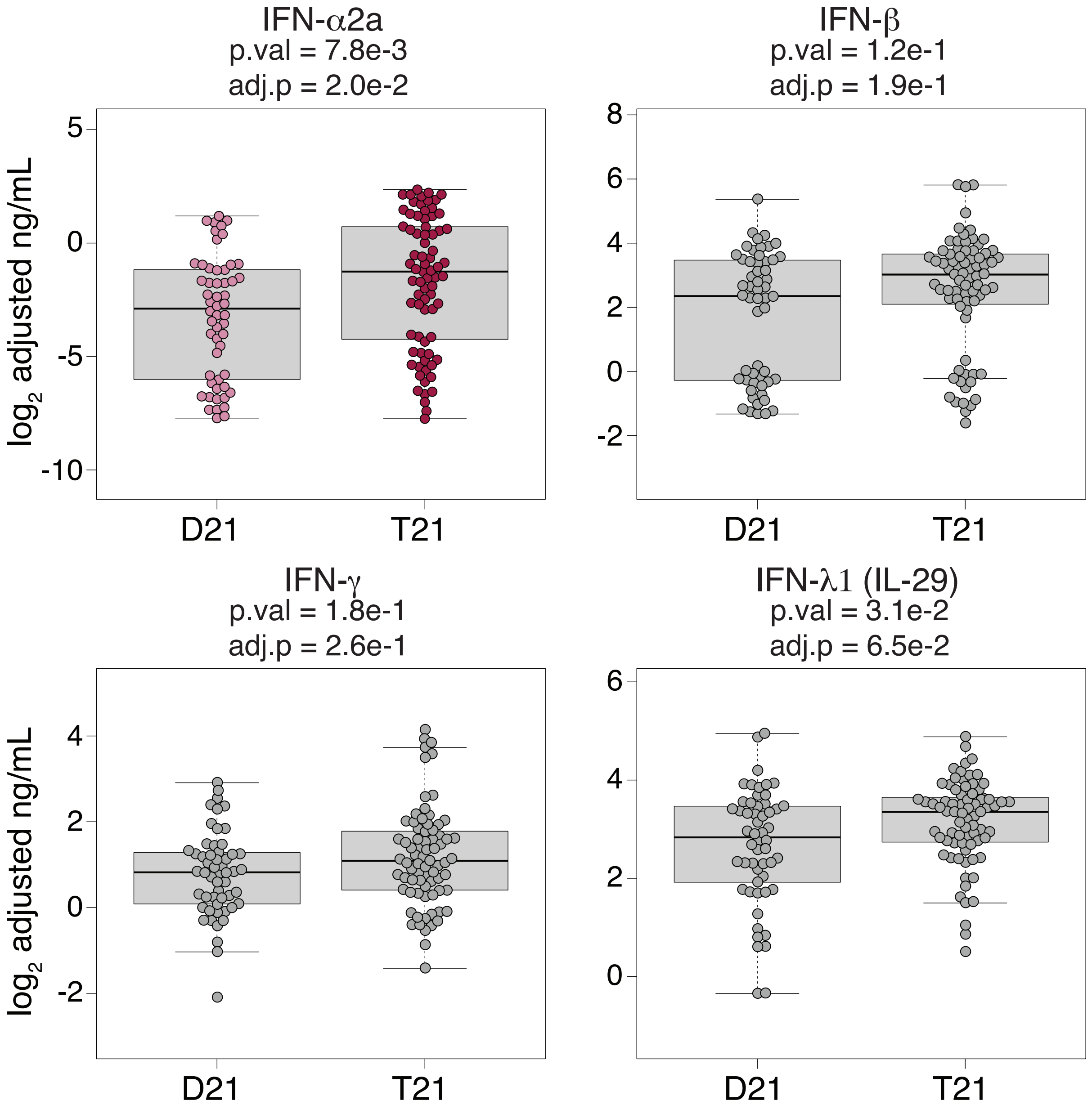

Supplementary Figure 3 – Levels of IFN ligands in people with and without Down syndrome. Boxplots of levels for four interferon ligands measured by multiplex MesoScale Discovery (MSD) assay in Cohort 3, n=128 independent samples, 74 with T21. Significant differences were assessed using the Kolmogorov-Smirnov test and an FDR-adjusted p-value

threshold of 0.05. All boxplots show median, 25th, and 75th percentile values. Error bars are 1.5 times the interquartile range (IQR) or the maximum data point if less than 1.5 IQR.

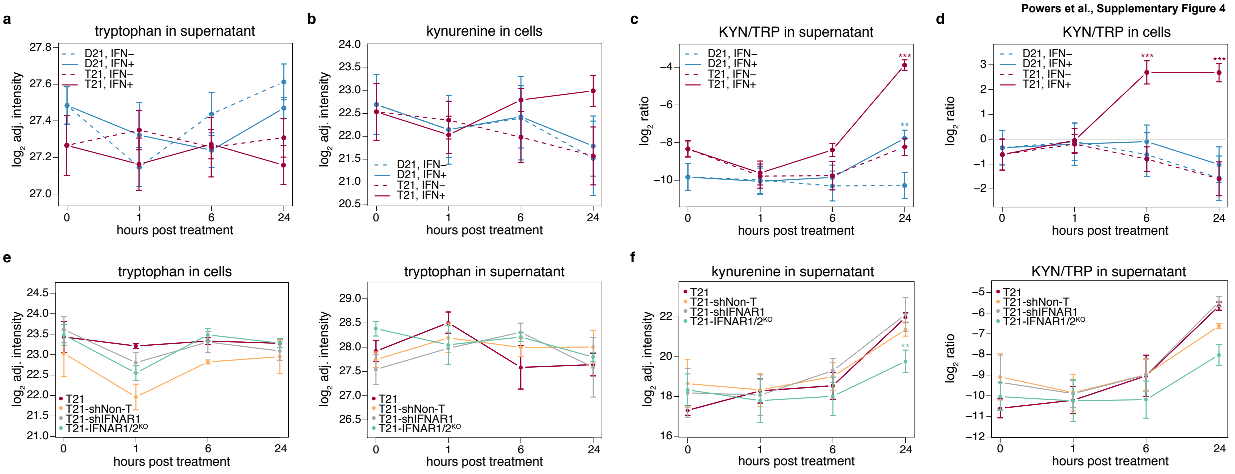

Supplementary Figure 4 – Trisomy 21 sensitizes cells to super-induction of the kynurenine pathway by IFN-α.

Metabolic tracing experiment using isotopologue-labeled ($^{13}C^{15}N$) tryptophan in fibroblast cell lines (n = 6, 3 with T21). **a)** Levels of isotopologue-labeled tryptophan in D21 and T21 supernatant, with or without IFN-α treatment. **b)** Levels of isotopologue-labeled kynurenine in D21 and T21 fibroblast cell lysates, with or without IFN-α treatment. **c)** Ratio of isotopologue-labeled kynurenine to isotopologue-labeled tryptophan levels in D21 and T21 fibroblast supernatants, with or without IFN-α treatment. **d)** Ratio of isotopologue-labeled kynurenine to isotopologue-labeled tryptophan levels in D21 and T21 fibroblast cell lysates, with or without IFN-α treatment. At each timepoint, the p-value between the untreated (IFN-) and IFN-treated (IFN+) samples was calculated using a two-tailed student's t-test (* $p < 0.05$, ** $p < 0.01$, *** $p < 0.001$). **e)** Levels of isotopologue-labeled tryptophan in the indicated cells and supernatants during a 24-hour time course of IFN-α treatment. **f)** Levels of isotopologue-labeled kynurenine in the supernatants of the indicated cell line and ratio of isotopologue-labeled kynurenine to isotopologue-labeled tryptophan levels in the supernatants of the indicated cell line during a 24-hour time course of IFN-α treatment. All experiments were performed in triplicate for a total of

n=18. At each time point, the p-value between the parental T21 cell line and each T21 cell line with modified *IFNAR* levels was calculated using a two-tailed student's t-test (* $p < 0.05$, ** $p < 0.01$, *** $p < 0.001$). Data shown in all panels are mean +/- standard error of the mean (SEM).
