# Supplementary material for: Trisomy 21 activates the kynurenine pathway via increased dosage of interferon receptors
Source: Nat Commun. 2019 Oct 18;10:4766. doi: 10.1038/s41467-019-12739-9 (PMC6800452; doi:10.1038/s41467-019-12739-9)
Supplement: Supplementary file 4 — Reporting Summary [file 41467_2019_12739_MOESM4_ESM.pdf]

nature research

Corresponding author(s): Kelly D. Sullivan, Joaquin M. Espinosa

Last updated by author(s): Sep 11, 2019

Reporting Summary

Nature Research wishes to improve the reproducibility of the work that we publish. This form provides structure for consistency and transparency in reporting. For further information on Nature Research policies, see Authors & Referees and the Editorial Policy Checklist.

Statistics

For all statistical analyses, confirm that the following items are present in the figure legend, table legend, main text, or Methods section.

n/a	Confirmed	
☐	☒	The exact sample size (*n*) for each experimental group/condition, given as a discrete number and unit of measurement
☐	☒	A statement on whether measurements were taken from distinct samples or whether the same sample was measured repeatedly
☐	☒	The statistical test(s) used AND whether they are one- or two-sided<br>*Only common tests should be described solely by name; describe more complex techniques in the Methods section.*
☐	☒	A description of all covariates tested
☐	☒	A description of any assumptions or corrections, such as tests of normality and adjustment for multiple comparisons
☐	☒	A full description of the statistical parameters including central tendency (e.g. means) or other basic estimates (e.g. regression coefficient) AND variation (e.g. standard deviation) or associated estimates of uncertainty (e.g. confidence intervals)
☐	☒	For null hypothesis testing, the test statistic (e.g. *F*, *t*, *r*) with confidence intervals, effect sizes, degrees of freedom and *P* value noted<br>*Give P values as exact values whenever suitable.*
☒	☐	For Bayesian analysis, information on the choice of priors and Markov chain Monte Carlo settings
☒	☐	For hierarchical and complex designs, identification of the appropriate level for tests and full reporting of outcomes
☐	☒	Estimates of effect sizes (e.g. Cohen's *d*, Pearson's *r*), indicating how they were calculated

Our web collection on statistics for biologists contains articles on many of the points above.

Software and code

Policy information about availability of computer code

Data collection	Metabolomics data were collected on a Vanquish UHPLC system coupled online to a Q Exactive mass spectrometer (Thermo Fisher). Mesoscale Discovery data were collected on a MESO QuickPlex SQ 120. RNAseq data were generated on a HiSeq4000. Q-RT-PCR data were collected on a Viia7 Real-Time PCR system (Life Technologies/Thermo Fisher Scientific). Western Blot images were captured on an ImageQuant LAS4000 (GE).
Data analysis	Metabolomic data were analyzed with MAVEN and Compound Discoverer (Thermo Fisher). Photoshop was used for western blot image processing. Q-RT-PCR data were analyzed in Prism. RNAseq data were analyzed using, TopHat2, HTSeq, and DESeq2. Custom data for other analyses are available at https://github.com/CostelloLab/Trisomy21_KYN_metabolomics.

For manuscripts utilizing custom algorithms or software that are central to the research but not yet described in published literature, software must be made available to editors/reviewers. We strongly encourage code deposition in a community repository (e.g. GitHub). See the Nature Research guidelines for submitting code & software for further information.

Data

Policy information about availability of data

All manuscripts must include a data availability statement. This statement should provide the following information, where applicable:

- Accession codes, unique identifiers, or web links for publicly available datasets
- A list of figures that have associated raw data
- A description of any restrictions on data availability

RNA-seq data were deposited at the Gene Expression Omnibus (National Center for Biotechnology Information) with series accession number GSE128622.
Metabolomics data were deposited in the Metabolomics Workbench database with Study IDs ST001240, ST001241, ST001242, and ST001243.
All data and R code developed for the full set of statistical metabolomic analyses is available at: https://github.com/CostelloLab/Trisomy21_KYN_metabolomics.

Field-specific reporting

Please select the one below that is the best fit for your research. If you are not sure, read the appropriate sections before making your selection.

☒ Life sciences ☐ Behavioural & social sciences ☐ Ecological, evolutionary & environmental sciences

For a reference copy of the document with all sections, see nature.com/documents/nr-reporting-summary-flat.pdf

Life sciences study design

All studies must disclose on these points even when the disclosure is negative.

Sample size	No sample-size calculations were performed. Sample size was determined to be adequate based on the magnitude and consistency of measurable differences between groups.
Data exclusions	Individuals with recent or active infections are excluded from our protocol.
Replication	For each series of experiments, all replication attempts were successful.
Randomization	Metabolomics samples were extracted and run with LC-MS in random order.
Blinding	Metabolomics sample preparation was done in a blinded fashion - sample labels (D21 or T21) were revealed post-measurement during the data analysis phase.

Reporting for specific materials, systems and methods

We require information from authors about some types of materials, experimental systems and methods used in many studies. Here, indicate whether each material, system or method listed is relevant to your study. If you are not sure if a list item applies to your research, read the appropriate section before selecting a response.

Materials & experimental systems

n/a	Involved in the study	
☐	☒	Antibodies
☐	☒	Eukaryotic cell lines
☒	☐	Palaeontology
☐	☒	Animals and other organisms
☐	☒	Human research participants
☒	☐	Clinical data

Methods

n/a	Involved in the study	
☒	☐	ChIP-seq
☒	☐	Flow cytometry
☒	☐	MRI-based neuroimaging

Antibodies

Antibodies used	All antibodies used are described in the methods section.
Validation	All information for each antibody including manufacturer and catalog number are included in the methods section.

Eukaryotic cell lines

Policy information about cell lines

Cell line source(s)	All cell lines were procured from the Coriell Cell repository.
Authentication	Genotypes were confirmed via Q-PCR.
Mycoplasma contamination	All cell lines were confirmed mycoplasma negative by PCR.
Commonly misidentified lines (See ICLAC register)	No commonly misidentified cell lines were used in this study.

Animals and other organisms

Policy information about studies involving animals; ARRIVE guidelines recommended for reporting animal research

Laboratory animals	Species strain, sex and age, are reported in the main text, methods, and Supplementary_Data_12_Mouse_Metabolomics_Data.

Wild animals	The study did not involve wild animals.
Field-collected samples	The study did not involve samples collected from the field.
Ethics oversight	All animal handling procedures were approved by the University of Colorado's Institutional Animal Care and Use Committee.

Note that full information on the approval of the study protocol must also be provided in the manuscript.

Human research participants

Policy information about studies involving human research participants

Population characteristics	Relevant population characteristics are included in Supplementary_Data_1_Cohort_Details.
Recruitment	Participants are recruited through a variety of mechanisms, including through local collaborating providers, advocacy and outreach groups, and community announcements.
Ethics oversight	All human subjects in this study were consented according to Colorado Multiple Institutional Review Board (COMIRB)- and Sant Pau Ethic Committee-approved protocols.

Note that full information on the approval of the study protocol must also be provided in the manuscript.
